# Epidemiology of Colorectal Cancer in Saudi Arabia: A Review

**DOI:** 10.7759/cureus.64564

**Published:** 2024-07-15

**Authors:** Ahmed M Alessa, Abdul Sattar Khan

**Affiliations:** 1 Saudi Board of Preventive Medicine, Community Health Wellness, Al-Ahsa, SAU; 2 Family and Community Medicine, King Faisal University, Al-Ahsa, SAU

**Keywords:** risk factors, survival rate, preventive measures, prevalence, incidence, colo-rectal cancer

## Abstract

Colorectal cancer (CRC) is the second leading cause of cancer death in the world, originating from the glandular epithelial cells of the large intestine and the rectum. This article aims to review the epidemiology of CRC in Saudi Arabia, focusing on prevalence, incidence, risk factors, preventive measures, and outcomes.

This narrative review utilized the PubMed database for data extraction, including freely accessible studies published in the last 15 years. Sixteen articles from different study designs were included, while awareness and non-English language studies were excluded.

In 2020, the incidence and mortality rate of CRC in Saudi Arabia were 14.6% and 1.48% among all cancers, respectively. From 2006 to 2016, the number of colon cancer and rectal cancer cases increased by 8% and 7%, respectively. Risk factors for CRC in Saudi Arabia include low education level, unemployment, physical inactivity, excess weight, poor knowledge of foods rich in fiber, cigarette smoking, reduced serum vitamin D and calcium levels, and certain gene mutations. National guidelines in Saudi Arabia recommend CRC screening for all individuals above 45 years using colonoscopy, flexible sigmoidoscopy, or fecal occult blood test. The 10-year survival rate for CRC in Saudi Arabia is 44.6%. The overall 5-year survival rate for the Ministry of National Guard-Health Affairs is 52.0%.

To lower the incidence and mortality of CRC, primary, secondary, and tertiary prevention are all very important. The most crucial aspect is to concentrate on primary prevention, which may involve raising public awareness of CRC risk factors and strategies for reducing or eliminating them.

## Introduction and background

Colorectal cancer (CRC) is the second leading cause of cancer death worldwide [1]. CRC usually originates from the glandular epithelial cells of the large intestine and the rectum [2,3]. Most CRC cases develop slowly from adenomatous polyps or adenomas [4]. Adenocarcinoma accounts for up to 95% of CRC cases, followed by carcinoid tumors, gastrointestinal stromal tumors (GISTs), lymphomas, and sarcomas [5].

CRC was the third most diagnosed malignant disease in 2020 in the world, with 1.93 million new cases (10% of total malignancies), after breast cancer with 2.26 million new cases (11.7% of total malignancies) and lung cancer with 2.21 million new cases (11.4% of total malignancies) [1]. CRC affects males more than females, with a cumulative risk of 2.71 and 1.83, respectively, and a male-to-female incidence rate ratio of 1.62 [1]. The highest incidence of CRC is in Australia and New Zealand (36.7 cases per 100,000), followed by Europe (28.8-32.1 cases per 100,000), Eastern Asia (26.5 cases per 100,000), and North America (26.2 cases per 100,000), while the lowest incidence is in Africa (6.4-9.2 cases per 100,000) and South-Central Asia (4.9 cases per 100,000) [3].

CRC risk factors are categorized as modifiable (changeable) and non-modifiable (unchangeable). Non-modifiable risk factors include aging (more common after age 50), history of colorectal polyps or CRC, inflammatory bowel disease (e.g., ulcerative colitis or Crohn’s disease), family history of CRC or adenomatous polyps, and hereditary diseases (e.g., pre-cancerous conditions) such as familial adenomatous polyposis and Lynch syndrome [6]. Modifiable risk factors include overweight and obesity, physical inactivity, dietary habits (e.g., high intake of red or processed meat, low intake of foods containing whole grains or dietary fiber, and diet low in dairy products), and behaviors like cigarette smoking and alcohol consumption [3].

Prevention of CRC can be achieved through primary, secondary, and tertiary prevention strategies. Primary prevention includes smoking cessation, a healthy diet, and regular exercise. Some chemopreventive agents can also reduce the risk of CRC development, such as higher intake and serum concentrations of vitamin D, higher intake of calcium, and regular intake of aspirin and non-steroidal anti-inflammatory drugs (NSAIDs). The US Preventive Services Task Force recommends the use of low-dose aspirin for the primary prevention of cardiovascular disease and CRC in adults aged 50-69 years. Secondary prevention includes screening methods such as sigmoidoscopy, colonoscopy, fecal occult blood test, or fecal immunochemical test (FIT). Tertiary prevention involves a healthy lifestyle and regular use of aspirin and other NSAIDs [7].

Although there are many studies on CRC in Saudi Arabia, comprehensive reviews are limited. Given its high prevalence and fatal nature, this study aims to review the comprehensive epidemiology of CRC in Saudi Arabia, including prevalence, incidence, risk factors, preventive measures, and outcomes.

## Review

Methods

**Figure 1:**
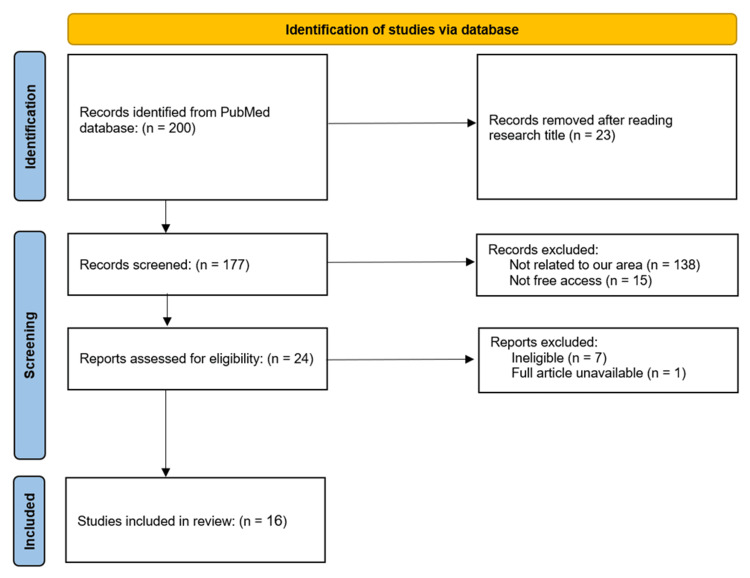
Flowchart of selection of the studies for review.

This narrative review aims to examine the epidemiology of CRC in Saudi Arabia, covering aspects such as prevalence, incidence, risk factors, preventive measures, and outcomes. The target population includes all residents of the Kingdom of Saudi Arabia. The literature search was conducted using the PubMed database with the following search terms: (colon cancer OR colorectal cancer) AND (incidence OR prevalence OR risk factors OR prevention OR outcomes) AND (Saudi Arabia). The inclusion criteria were studies published in the last 15 years (from 2009 to 2023), freely accessible studies, those addressing the specified aspects of CRC within the Saudi Arabian context, and English-language studies only. Exclusion criteria included studies focused on awareness campaigns and non-English language studies. The study designs considered were case reports, case series, case-control studies, cohort studies, and systematic reviews. This structured approach ensures a comprehensive and relevant collection of data to understand the epidemiology of CRC in Saudi Arabia. Our search ended up with 16 articles that matched our inclusion and exclusion criteria (Figure 1).

Results

The results of this review are summarized in Table 1.

**Table 1:** Summary of studies of colorectal cancer in Saudi Arabia. CRC: colorectal cancer.

Reference	Year	Study design	Sample size	Main relevant findings
Incidence and mortality				
Alsanea et al. [8]	2015	Retrospective analysis	549	The age-standardized incidence rate and percentage of CRC cases of all diagnosed cancers were increased by twofold from 1994 to 2010
Almatroudi et al. [9]	2016	Retrospective observational population-based study	13,013	From 2006 to 2016, the number of colon cancer and rectal cancer cases increased by 8% and 7%, respectively
Alqahtani et al. [10]	2020	Systematic review and meta-analysis	39 full-text records 17,522 cases	The incidence and mortality rate of CRC in Saudi Arabia were 14.6% (cumulative risk 1.47%) and 1.48% (cumulative risk 0.65%) among all cancers, respectively
Risk factors				
Almurshed et al. [11]	2009	Case-control study	100	Low education level, unemployment, not taking exercise regularly, and poor knowledge of high-fiber diets were significant risk factors for CRC
Shalaby et al. [12]	2014	Case-control study	250	Matrix metalloproteinase-2 C-1306 T polymorphism is significantly more common in colon cancer patients than general Saudi population
Khabaz et al. [13]	2016	Case-control study	118	Deletion of glutathione S-transferase Mu 1 gene showed a significant association with colorectal malignancies
Karam et al. [14]	2016	Case-control study	200	The frequencies of the GG genotype of X-ray repair cross-complementing protein 1399 polymorphism were significantly higher in the CRC patients than in normal individuals
Alhadheq et al. [15]	2016	Case-control study	373	Single nucleotide polymorphisms rs8679 showed decreased susceptibility to CRC at heterozygous TC allele and at minor allele C
Elzein et al. [16]	2017	Case report study	1	Silent CRC associated with *Streptococcus bovis* endocarditis in a patient who underwent prosthetic valve replacement
Al-Zalabani et al. [17]	2019	Retrospective analysis	1465	Physical inactivity was linked to the largest fraction of attributable colorectal cases in Saudi Arabia, with a similar population-attributable fraction among men (16.13%) and women (16.45%)
Al-Ghafari et al. [18]	2019	Case-control study	100	A decrease in serum vitamin D and calcium levels is significantly associated with CRC
AlMutairi et al. [19]	2019	Case-control study	274	Prostate cancer-associated noncoding RNA 1 single nucleotide polymorphisms rs1456315 had a statistically significant risk association with Saudi CTC patients
Rasool et al. [20]	2021	Case series study	100	Results revealed six mutations in 14 patients with CRC
Preventive measures				
Alsanea et al. [21]	2015	Systematic review and meta-analysis	12 full-text records	The panel recommends screening for CRC in Saudi Arabia in asymptomatic Saudi patients at average risk of CRC
Outcomes				
Alsanea et al. [8]	2015	Retrospective analysis	549	The overall five-year survival was 44.6% for the period from 1994 to 2004
Aldiab et al. [22]	2016	Longitudinal prospective analysis study	175	CRC in Saudi Arabia is usually diagnosed at advanced stages with metastases and is associated with poor prognosis and short survival
Alyabsi et al. [23]	2021	Retrospective cohort study	1012	The overall five-year survival for the Ministry of National Guard–Health Affairs was 52.0%

Incidence and Mortality Rates

**Figure 2:**
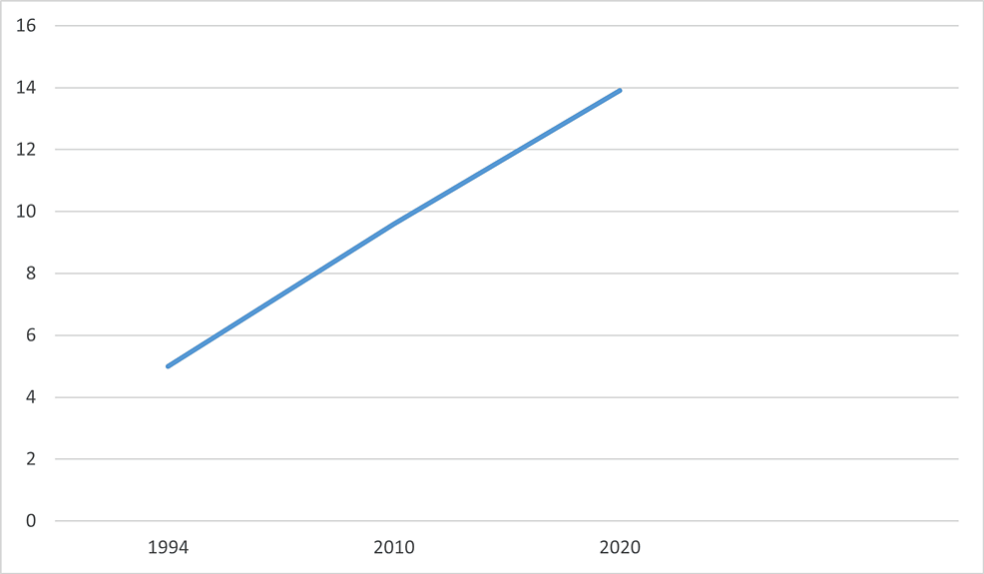
Age-standardized incidence rate trends of colorectal cancer in Saudi Arabia. Data from [8,10].

**Figure 3:**
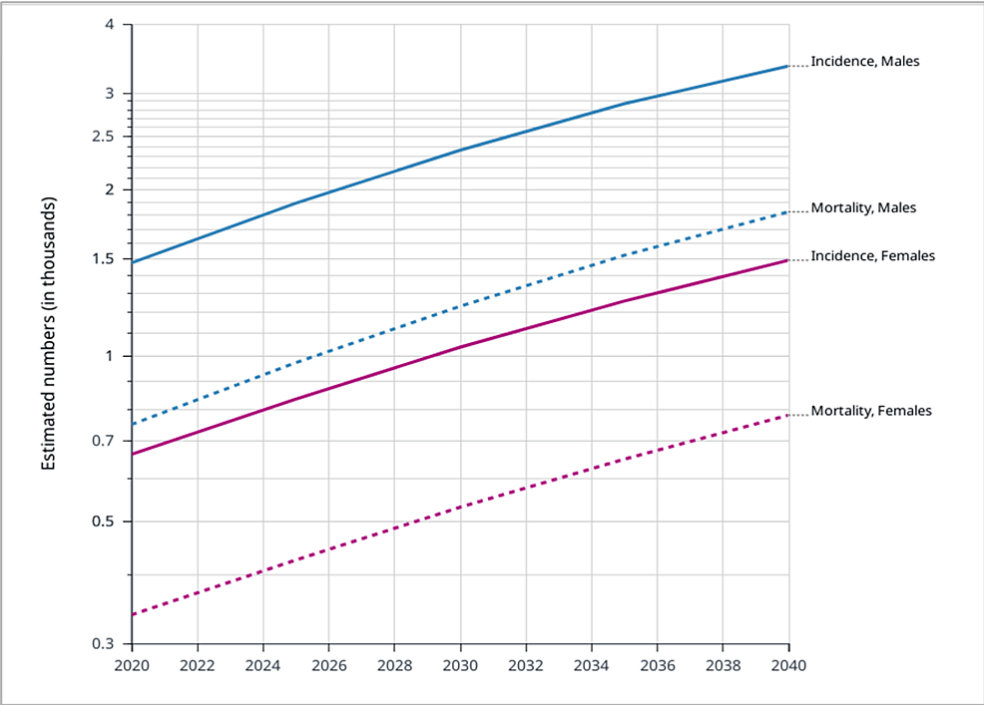
Estimated numbers of colorectal cancer in Saudi Arabia from 2020 to 2040: males and females per 100,000 people. Data from [24].

Among all cancers in Saudi Arabia, CRC had an incidence rate of 14.6% (cumulative risk 1.47%), with 19.6% in males and 9.5% in females, just second to breast cancer with an incidence of 14.8% (cumulative risk 2.87%) in 2020. From 2006 to 2016, the number of CRC cases increased, with an 8% increase in colon cancer and a 7% increase in rectal cancer. From 1994 to 2010, the age-standardized incidence rate (ASIR) nearly doubled from 5.0 to 9.6 per 100,000. Over the same period, the percentage of CRC cases among all diagnosed cancers doubled from 4.8% to 10.1%. Figure 2 shows the increasing trend of ASIR in Saudi Arabia from 1994 to 2020 [8-10]. The percentage of mortality of CRC among all diagnosed cancers in Saudi Arabia was 1.48% with a cumulative risk of 0.65%. The highest mortality rates were for breast cancer, leukemia, and liver cancer, with percentages of 8.5%, 8.7%, and 8.7%, respectively. Figure 3 shows the estimated cumulative incidence rate (CIR) and crude mortality rate (CMR) in Saudi Arabia from 2020 to 2040. Table 2 shows the incidence and mortality rates of CRC in Gulf Cooperation Council Countries according to the International Agency for Research (IAR) on Cancer in 2020 [10,24].

**Table 2:** Incidence and mortality rates of colorectal cancer in Gulf Cooperation Council Countries [24]. CIR: crude incidence rate, ASIR: age-standardized incidence rate, ICR: Incheon Cancer Registry, CMR: crude mortality rate, ASMR: age-standardized mortality rate, MCR: mortality-to-case ratio.

Gulf country	CIR	ASIR	ICR	CMR	ASMR	MCR
Bahrain	8.6	13.9	3.56	3.8	7.1	2.28
Kuwait	9.6	12.5	3.41	4.4	6.6	2.31
Oman	7.5	9.9	1.69	4.2	5.7	1.08
Qatar	6.0	15.7	6.38	2.8	9.0	4.63
Saudi Arabia	11.5	13.9	3.07	5.7	7.3	1.99
United Arab Emirates	5.2	13.1	4.61	2.2	6.9	3.26

Risk Factors

Some of the modifiable risk factors for CRC in Saudi Arabia relate to sociodemographic and lifestyle factors. A low education level is significantly associated with CRC (OR = 8.3, P < 0.05). Employment status is also significant, with CRC cases being more likely to be unemployed (OR = 3.7, P < 0.05). Physical inactivity plays a major role in CRC risk, with 16.13% of male and 16.45% of female CRC cases attributed to physical inactivity. CRC patients are more likely to engage in light exercise rather than moderate to heavy exercise (OR = 8.5, P < 0.05). Excess weight is another significant risk factor, with 9.71% of male cases and 6.93% of female cases attributed to obesity, and 6.05% of male cases and 1.9% of female cases attributed to being overweight. CRC patients also have significantly poorer knowledge of foods rich in fiber compared to those without CRC (OR = 17, P < 0.05). The risk of CRC among smokers and former smokers is higher in men, with 3.04% of male cases attributed to current smokers and 3.29% to former smokers, compared to 0.18% and 0.12%, respectively, in women [11,17]. Certain genotypes of the vitamin D receptor (VDR), specifically ApaI and TaqI gene polymorphisms, are linked with serum total vitamin D and calcium levels in CRC patients. The homozygous genotype (aa) of the ApaI VDR polymorphism (rs7975232) is correlated with total serum vitamin D levels, while the heterozygous (Tt) TaqI VDR polymorphism (rs731236) is associated with serum calcium levels [18].

Non-modifiable risk factors are related to genetics. A single nucleotide transition of the gene matrix metalloproteinase-2 (MMP2) at C-1306 T is significantly more common in CRC patients than in the general Saudi population (OR = 2.04, P = 0.0121). Deletion of the Glutathione S-Transferase Mu 1 (GSTM1) gene is significantly associated with CRC (OR = 2.571, P = 0.03706). Polymorphism in the GG genotype of X-ray repair cross-complementing protein (XRCC1) 399 is also a significant risk factor (OR = 2.1, P = 0.03). Single nucleotide polymorphism rs1456315 in LncRNA prostate cancer non-coding RNA (PRNCR1) is significantly associated with CRC in the homozygous variant "CC" genotype (OR = 2.09, P = 0.02), minor allele "C" (OR = 1.55, P = 0.01), and additive genotype "TC+CC" (OR = 1.64, P = 0.04). A case series study reveals six BRAF gene mutations in 14 CRC cases out of 100 patients, including c.1799 T > A (V600E), c.1758delA, c.1860insA/C, c.1780G > A; D594N, c.1826insT, and c.1860insA. Conversely, single nucleotide polymorphism rs8679 in poly (ADP-ribose) (PARP-1) polymerase shows decreased susceptibility to CRC in the heterozygous TC allele (OR: 0.56, P < 0.009) and in the minor allele C (OR = 0.695, P = 0.02927) [12-15,19,20].

A case reported that a 75-year-old woman who underwent prosthetic mitral valve replacement developed *Streptococcus bovis* endocarditis and silent invasive colorectal adenocarcinoma after undergoing screening by colonoscopy one year later. This case highlights the association between *Streptococcus bovis* endocarditis and CRC [16].

Preventive Measures

The only preventive measure recommended in Saudi Arabia is screening. The national guidelines for CRC screening in Saudi Arabia strongly recommend screening all individuals above the age of 45 years with low quality of evidence. Screening for persons over 70 years is suggested only conditionally and with low evidence, unless they are healthy, lack comorbidities, and have a life expectancy greater than 10 years at the time of screening. It is strongly recommended to screen for CRC using colonoscopy or flexible sigmoidoscopy, with low and moderate quality evidence, respectively. Colonoscopy is conditionally recommended over colonography for screening purposes (low-quality evidence). Additionally, flexible sigmoidoscopy is recommended over the guaiac fecal occult blood test (very low-quality evidence), though this recommendation is conditional. Lastly, colonoscopy is conditionally recommended over flexible sigmoidoscopy for screening (low-quality evidence) [21].

Outcomes

The outcomes of CRC can include metastases, treatment, recurrence, or death. In 2016, a study found that 47.73% of CRC patients had metastases detected at enrollment. Metastases were found in 52.38% of lymph nodes, 31.33% of livers, 7.94% of lungs, and 6.35% of peritoneum. Out of 175 CRC cases, distant metastases occurred in 4 patients with stage IIC, 10 patients with stage IIIA, 11 patients with stage IIIB, 13 patients with stage IIIC, and 14 patients with stage IV tumors. Data on treatment were limited and are to be studied further. Recurrence occurred in 29.71% of CRC patients after initial management (23 males and 29 females, P = 0.0468) [22].

Comparing CRC patients with or without KRAS and BRAF gene mutations, a significant difference was found in time to recurrence. The median time to recurrence in patients with KRAS mutations was 34 months (95% CI: 32-40 months), compared to 53 months (95% CI: 44-58 months) in those without mutations. For BRAF mutations, the median time to recurrence was 17 months (95% CI: 15-19 months), compared to 52 months (95% CI: 50-56 months) in those without mutations. A significant difference in survival rates was found between patients with or without KRAS and BRAF mutations (log-rank test: χ² = 72.2542, p < 0.0001 for KRAS; χ² = 59.9886, p < 0.0001 for BRAF). The median survival for patients with KRAS mutations was 34 months (95% CI: 31-38 months), compared to 54 months (95% CI: 48-58 months) in those without mutations. For BRAF mutations, the median survival was 21 months (95% CI: 18-28 months), compared to 52 months (95% CI: 48-58 months) in those without mutations. Table 3 summarizes the prognostic factors for recurrence and survival in CRC patients. Another study assessed the survival of CRC patients in Saudi Arabia from 1994 to 2004. The 5-year survival rate for CRC was 44.7% for 1994-1999 and 44.3% for 2000-2004. The 10-year survival rate for both periods was 44.6% [8,22]. According to the Ministry of National Guard-Health Affairs registry data, the survival rates of 1012 CRC patients diagnosed between 2009 and 2017 are 83% for one year, 65% for three years, and 52.0% for five years. The five-year survival rates for localized stage, regional stage, and distant metastases are 79.85%, 63.25%, and 20.31%, respectively [23].

**Table 3:** Prognostic factors for recurrence and survival in colorectal cancer patients [22]. RR: relative risk, CRC: colorectal cancer.

Parameter	P value	RR
CRC recurrence		
Stage of disease at diagnosis (stage III/IV)	0.026	1.38
Lymphovascular invasion	0.048	1.21
Lymph node involvement	0.0383	1.04
Metastases	0.0241	1.41
KRAS mutations	0.0413	1.53
BRAF mutations	0.002	1.83
Survival		
Lifestyle patterns	0.033	1.01
Location of the primary tumor	0.047	1.16
Obesity	0.043	1.54
Stage of disease at diagnosis (stage III/IV)	0.047	1.56
Tumor histopathology	0.026	1.11
Lymphovascular invasion	0.000	1.75
Distant metastases	0.042	1.07
KRAS mutations	0.000	1.09
BRAF mutations	0.033	1.10

Discussion

According to IAR on Cancer in 2020, CRC is the second leading cancer in Saudi Arabia, with a crude incidence rate (CIR) of 11.5 per 100,000, 13.7 for males and 8.5 for females. The age-standardized incidence rate (ASIR) was 13.9 with a cumulative risk of 3.07%, second only to breast cancer, which had a CIR of 26.9 and an ASIR of 28.8 with a cumulative risk of 4.38 per 100,000. The ASIR of CRC in Saudi Arabia is much lower than in developed countries such as the United States (25.6), United Kingdom (34.1), Germany (25.8), France (30.1), and Japan (38.5). Compared to Gulf Cooperation Council countries, Saudi Arabia had the highest CIR and the second-highest ASIR after Qatar (15.7) [24]. Saudi Arabia also had the highest crude mortality rate (CMR) among Gulf Cooperation Council countries at 5.7, and the age-standardized mortality rate (ASMR) was 7.3 per 100,000 with a cumulative risk of 1.99% in 2020 [24].

Obesity, overweight, and physical inactivity are some of the most modifiable risk factors for CRC that can be prevented [3,6]. It is reported that 38% of the Saudi population are overweight, and 20% are obese, while the percentage of physical inactivity is 80% [25]. For an adult to be physically active, they need to practice 150 minutes of moderate-intensity physical activity, 75 minutes of vigorous-intensity physical activity, or an equivalent combination of moderate- and vigorous-intensity activity each week [26]. Along with our review, multiple pieces of evidence support that cigarette smoking is associated with the development of CRC [6,27,28]. Some studies report that reduced serum vitamin D and calcium levels increase the risk of CRC, while other studies do not [29,30]. Klampfer L. reviewed that dietary vitamin D3 intake or sunlight exposure has an inverse association with CRC [30]. Additionally, calcium intake has been shown to reduce the risk of developing CRC and improve the survival rate [31-33].

Saudi guidelines for CRC screening strongly recommend screening individuals between 45 and 70 years of age. In comparison, the United States Preventive Services Task Force (USPSTF) strongly recommends screening individuals between 50 and 75 years of age, and those between 45 and 49 years with moderate net benefit [34]. The Saudi guidelines do not recommend screening individuals over 70 years old unless the person could benefit from the screening. Similarly, the USPSTF conditionally recommends screening for adults between 76 and 85 years [34].

Limitation

More research could be included if multiple database searches were conducted rather than relying on a single database. Additional keywords, such as "neoplasia," "adenocarcinoma," and "carcinoma predictors," could also be added to the search. The studies included were only in English and published in the last 15 years.

## Conclusions

Given that CRC is the second most common cancer in Saudi Arabia, the Ministry of Health will need to invest more funds and treat a larger number of patients. Therefore, it is crucial to prioritize cancer prevention, particularly primary prevention, over secondary and tertiary prevention. Increasing public knowledge of CRC risk factors and strategies for avoiding or managing them could be the first step in the primary prevention of CRC. Also, prevention of socioeconomic factors is very important which could be done by physical activity and healthy diet. The role that calcium and vitamin D intake play in preventing CRC still requires further investigation. Regular publications of updated CRC data, including incidence, mortality, and survival rates, are warranted.
